# Supplementary material for: Identification of photoperiod-induced specific miRNAs in the adrenal glands of Sunite sheep (Ovis aries)
Source: Front Vet Sci. 2022 Jul 22;9:888207. doi: 10.3389/fvets.2022.888207 (PMC9354845; doi:10.3389/fvets.2022.888207)
Supplement: Supplementary file 1 [file Data_Sheet_1.ZIP › Supplementary file/miRDeep2 file.zip!/miRDeep2 file/Know_Structure/SP42A2/oar-mir-362.pdf]

miRBase precursor	: oar-mir-362
Total read count	: 1435
oar-miR-362 read count	: 1407
remaining reads	: 28

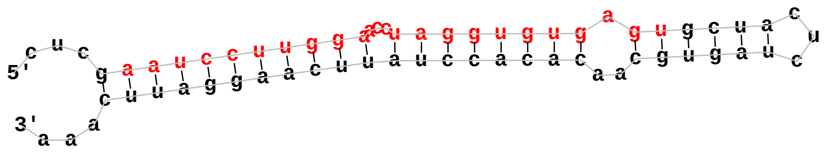

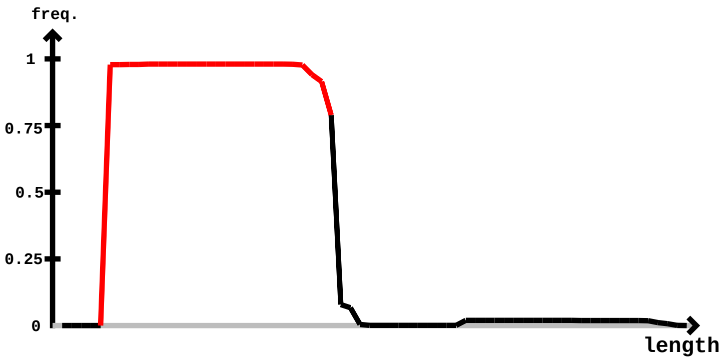


```
       oar-miR-362
5'- cucgaauccuuggaaccuaggugugagugcuacucuagugcaacacaccuauucaaggauucaaa   -3'    exp
    ...(((((((((((...((((((((.((((((...))))))..)))))))))))))))))))...    reads      mm         sample
    ....aauccuuggaaccuaggug..........................................    1          0          seq
    ....aauccuuggaaccuaggugu.........................................    4          0          seq
    ....aauccuuggaaccuaggugug........................................    51         0          seq
    ....aauccuuggaaccuagguguga.......................................    37         0          seq
    ....aauccuuggaaccuaggugugag......................................    181        0          seq
    ....aauccuuggaaccuaggugugagu.....................................    1017       0          seq
    ....aauccuuggaaccuaggugugagug....................................    17         0          seq
    ....aauccuuggaaccuaggugugagugc...................................    91         0          seq
    ....aauccuuggaaccuaggugugagugcu..................................    5          0          seq
    ......uccuuggaaccuaggugugagu.....................................    1          0          seq
    ........cuuggaaccuaggugug........................................    1          0          seq
    ........cuuggaaccuaggugugagu.....................................    1          0          seq
    ..............................uacucuagugcaacacaccuauu............    1          0          seq
    .........................................aacacaccuauucaaggau.....    1          0          seq
    .........................................aacacaccuauucaaggauu....    10         0          seq
    .........................................aacacaccuauucaaggauuc...    6          0          seq
    .........................................aacacaccuauucaaggauuca..    9          0          seq
    .........................................aacacaccuauucaaggauucaa.    1          0          seq
```
